# IMPACT OF QUARANTINE AND INFODEMIC DUE TO THE COVID-19 PANDEMIC ON MENTAL HEALTH: EXPERIENCE of UKRAINE

**DOI:** 10.1192/j.eurpsy.2023.1692

**Published:** 2023-07-19

**Authors:** M. Markova, N. Maruta, A. Markov, T. Aliieva, T. Abdriakhimova

**Affiliations:** 1 Sexology, Psychotherapy and Medical Psychology, Kharkiv Medical Academy of Postgraduate Education; 2 SI “Institute of Neurology, Psychiatry and Narcology of the NAMS of Ukraine”, Kharkiv; 3 Research Institute of Psychiatry Ministry of Health of Ukraine” SI; 4 Bogomolets National Medical University, Kyiv, Ukraine

## Abstract

**Introduction:**

Today the psycho-traumatic impact of quarantine due to the COVID-19 pandemic and the infodemic, as a separate psycho-traumatic factor, on mental health remain unclear

**Objectives:**

To study the impact of quarantine and infodemic due to the COVID-19 pandemic on the mental health of the population of Ukraine

**Methods:**

During quarantine 902 Ukrainian people voluntarily completed the questionnaire in Google format a questionnaire containing psychodiagnostic tools for assessing the level of stress L. Reeder, anxiety response GAD-7, depression PHQ-9, strategies for stress-coping behavior E. Heim, vitality S. Maddy and developed by us based on the AUDIT Test for the detection of disorders related to the obsession with news associated with the COVID-19 pandemic

**Results:**

The investigations carried out suggested that the quarantine restrictions could be predisposing factors for mental health impairments. The threat of coronavirus disease, a disruption of a habitual life stereotype, leisure restrictions, a harmful interest in news about the pandemic, and tobacco abuse play an important role in mechanisms of distress formation. The analysis of examination of the quality of life has demonstrated, that 8.14 % had a low level of its physical component, 15.55 % had a low level of its emotional component, and 10.21 % had a low level of a social activity. Under these conditions, risks of increased stress pressure, anxiety, and depression are rising. During the quarantine period, 10.53% recorded a high level of stress, 11.75% had an average level of anxiety, 7.43% had a high level of anxiety, 9.53% had severe depression, and 7.76% had very severe depression. 5.93% were obsessed with information related to the pandemic, at the level of “addiction”, 9.95% “use” information with “harmful consequences”, 20.14% a “risky” level. A low level of vitality had a direct correlation with an excessive interest in news and the use of maladaptive strategies of the types of ignoring, dissimulation and confusion (cognitive), submissiveness, suppression of emotions and self-blame (emotional), avoidance (behavioral) - that is, passive forms of solving problems due to despair in one’s own strength and intellectual resources, with a deliberate underestimation of troubles

**Conclusions:**

Measures on psycho-prevention should be performed on the base of the regularities identified

**Disclosure of Interest:**

None Declared

